# Heterozygous knockout of Synaptotagmin13 phenocopies ALS features and TP53 activation in human motor neurons

**DOI:** 10.1038/s41419-024-06957-3

**Published:** 2024-08-03

**Authors:** Johannes Lehmann, Amr Aly, Christina Steffke, Luca Fabbio, Valentin Mayer, Natalie Dikwella, Kareen Halablab, Francesco Roselli, Simone Seiffert, Tobias M. Boeckers, David Brenner, Edor Kabashi, Medhanie Mulaw, Ritchie Ho, Alberto Catanese

**Affiliations:** 1 Institute of Anatomy and Cell Biology, Ulm University School of Medicine, Ulm, Germany; 2 Department of Neurology, Ulm University School of Medicine, Ulm, Germany; 3 German Center for Neurodegenerative Diseases (DZNE), Ulm Site, Ulm, Germany; 4 Institute of Human Genetics, Ulm University and Ulm University Medical Center, Ulm, Germany; 5 Institut Imagine, University Paris Descartes, Necker-Enfants Malades Hospital, Paris, France; 6 Unit for Single-Cell Genomics, Medical Faculty, Ulm University, Ulm, Germany; 7 Center for Neural Science and Medicine, Cedars-Sinai Medical Center, Los Angeles, CA USA; 8 Board of Governors Regenerative Medicine Institute, Cedars-Sinai Medical Center, Los Angeles, CA USA; 9 Department of Biomedical Sciences, Cedars-Sinai Medical Center, Los Angeles, CA USA; 10 Department of Neurology, Cedars-Sinai Medical Center, Los Angeles, CA USA

**Keywords:** Amyotrophic lateral sclerosis, Cell death

## Abstract

Spinal motor neurons (MNs) represent a highly vulnerable cellular population, which is affected in fatal neurodegenerative diseases such as amyotrophic lateral sclerosis (ALS) and spinal muscular atrophy (SMA). In this study, we show that the heterozygous loss of *SYT13* is sufficient to trigger a neurodegenerative phenotype resembling those observed in ALS and SMA. SYT13^+/−^ hiPSC-derived MNs displayed a progressive manifestation of typical neurodegenerative hallmarks such as loss of synaptic contacts and accumulation of aberrant aggregates. Moreover, analysis of the SYT13^+/−^ transcriptome revealed a significant impairment in biological mechanisms involved in motoneuron specification and spinal cord differentiation. This transcriptional portrait also strikingly correlated with ALS signatures, displaying a significant convergence toward the expression of pro-apoptotic and pro-inflammatory genes, which are controlled by the transcription factor TP53. Our data show for the first time that the heterozygous loss of a single member of the synaptotagmin family, *SYT13*, is sufficient to trigger a series of abnormal alterations leading to MN sufferance, thus revealing novel insights into the selective vulnerability of this cell population.

## Introduction

Spinal motor neurons (MNs) are a specialized neuronal population localized in the ventral horn of the spinal cord and responsible for the innervation and contraction of skeletal muscles. Loss of MNs is the main pathological aspect characterizing (among others) amyotrophic lateral sclerosis (ALS), a lethal and genetically heterogeneous neurodegenerative disease [1]. Despite our understanding of the complex genetic component of this pathology is increasing [2–4], the biological alterations determining the selective vulnerability of these cells have still to be fully clarified. Previous work aimed at elucidating the molecular principles of neuronal sufferance in ALS highlighted important anatomical and electrophysiological differences across the MN subtypes. On one side, slow (S) MNs appear to be small, have reduced dendritic arborization and high intrinsic excitability [5, 6]. Interestingly, these characteristics define the most disease-resistant MNs both in *post-mortem* samples and murine models of ALS [7–9]. In contrast, fast fatigable (FF) MNs, which have larger size and dendritic complexity, appear to degenerate already before symptoms onset [9, 10]. The increased vulnerability of this specific cellular population has been molecularly linked to increased ER stress, altered unfolded protein response (UPR), dysregulated calcium buffering, as well as altered synaptic plasticity and composition [9, 11–13]. These findings have been also replicated in experiments contrasting the transcriptomes of iPSC-derived vulnerable MNs and resistant oculomotor neurons (OMNs) [14], which highlighted transcriptional peculiarities that contribute in determining MN vulnerability or resistance in ALS. On the same line, an elegant work from Nizzardo and colleagues revealed that OMNs preferentially express Synaptotagmin 13 (SYT13) compared to spinal MNs [15]. In this study, the authors also found that the MNs still surviving within the spinal cord of end-stage ALS patients (most likely S type) had higher *SYT13* mRNA levels compared to those found in non-affected controls, suggesting that this protein might help in protecting MNs during disease progression. Accordingly, overexpression of SYT13 in ALS and SMA models proved neuroprotective. SYT13 is a non-redundant [16], atypical synaptotagmin missing a Ca^2+^-binding domain [17] whose function is still not fully understood. Despite previous studies have highlighted important aspects of selective MN vulnerability, the question whether the reduction of *SYT13* expression might be sufficient to trigger MN sufferance remains unanswered. To address this question, we created *SYT13* heterozygous knock-out hiPSCs (based on a parental line from a healthy donor) and differentiated them into spinal MNs. Notably, the partial loss of *SYT13* expression resulted to be sufficient in inducing some pathological phenotypes typically observed in ALS, thus highlighting novel and previously unappreciated features of this gene in MN vulnerability.

## Material and methods

### Human iPSCs

The SYT13^+/−^ hiPSCs were generated starting from the commercially-available PGP1 (GM23338) line. Guide RNA with the sequence GUGCAUGUGCCGACACAGGC and Cas9 were delivered to the cells by electroporation. Cells were then let recover in culture for 2 days before assessing the genome editing by Sanger sequencing after PCR amplification using the primers CAAAGATCCACGACCGCCT (forward) and AGCCTCCTCTGACGTCCTC (reverse). Single cells were then seeded for clonal expansion.

To minimize the possibility that the observed changes in SYT13^+/−^ hiPSCs might arise from the procedure of genetic engineering, the parental line PGP1 was also mock-transfected with Cas9 (referred to as WT or SYT13^+/+^ in the manuscript) following the same protocol.

HiPSCs were cultured on Matrigel^®^-coated (Corning, 354,277) 6-well plates at 37 °C (5% CO_2_, 5% O_2_) using mTeSR plus medium (Stem Cell Technologies, 100-0274). After reaching around 80% confluence, the colonies were detached using Dispase (Stem Cell Technologies, 07923) and passaged in a 1:3 or 1:6 split ratio. Potential mycoplasma contamination was regularly checked using the MycoStrip™ Mycoplasma Detection Kit (Invivogen, rep-mysnc-50; every second week) and MycoAlert^®^ Mycoplasma Detection Kit (Lonza, LT07-318; once a month).

### Differentiation of hiPSC into spinal motor neurons

We differentiated MNs from hiPSCs using a previously described protocol [18]. Briefly, hiPSC colonies were detached using Dispase (Stem Cell Technologies, 07923) and cultured in suspension in ultra-low attachment flasks T75 for 3 days for the formation of embryoid bodies (EBs) in hESC medium (DMEM/F12 + 20% knockout serum replacement + 1% NEAA + 1% β-mercaptoethanol + 1% antibiotic–antimycotic + SB-431542 10 µM + Dorsomorphin 1 µM + CHIR 99021 3 µM + Purmorphamine 1 µM + Ascorbic Acid 200 ng/µL + 1% B27 + 0.5% N_2_). On the fourth day, the medium was switched to MN Medium (DMEM/F12 + 24 nM sodium selenite + 16 nM progesterone + 0.08 mg/mL apotransferrin + 0.02 mg/mL insulin + 7.72 μg/mL putrescine + 1% NEAA, 1% antibiotic–antimycotic + 50 mg/mL heparin + 10 μg/mL of the neurotrophic factors BDNF, GDNF, and IGF-1, SB-431542 10 µM, Dorsomorphin 1 µM, CHIR 99021 3 µM, Purmorphamine 1 µM, Ascorbic Acid 200 ng/µL, Retinoic Acid 1 µM, cAMP 1 µM, 1% B27, 0.5% N_2_). After 5 further days of cultivation EBs were dissociated into single cells with Accutase (Sigma Aldrich) for 10 min and plated onto μDishes, 24-well μPlates (Ibidi) or 6-well plates (Corning) pre-coated with Growth Factor Reduced Matrigel (Corning).

### RNA sequencing

Messenger RNA was purified from total RNA using poly-T oligo-attached magnetic beads. After fragmentation, the first strand cDNA was synthesized using random hexamer primers, followed by the second strand cDNA synthesis using either dUTP. The library was ready after end repair, A-tailing, adapter ligation, size selection, USER enzyme digestion, amplification, and purification. The library was checked with Qubit and real-time PCR for quantification and bioanalyzer for size distribution detection. Quantified libraries will be pooled and sequenced on Illumina platforms, according to effective library concentration and data amount. Raw data (raw reads) in fastq format was firstly processed through in-house perl scripts. In this step, clean data (clean reads) was obtained by removing reads containing adapter, reads containing ploy-N and low-quality reads from raw data. At the same time, Q20, Q30 and GC content were calculated. All the downstream analyses were based on clean data with high quality. Reference genome and gene model annotation files were downloaded from genome website directly. Index of the reference genome was built using Hisat2 v2.0.5 and paired-end clean reads were aligned to the reference genome using Hisat2 v2.0.5. FeatureCounts v1.5.0-p3 was used to count the reads numbers mapped to each gene, followed by FPKM calculation of each gene.

### Western blot

Western blot experiments were performed by loading 10 μg of protein (determined by Bradford Assay) on 8% acrylamide gels, which were then transferred to a nitrocellulose membrane using a Trans-Blot Turbo device (BioRad, USA). To block non-specific binding sites, the membranes were incubated with a 5% BSA solution (diluted in TBS pH 7.5 + 0.2% TWEEN) for 2 h and incubated with the primary antibody overnight at 4 °C. Afterwards, blots were washed 3 times for 10 min with TBS + 0.2% TWEEN, incubated with HRP-conjugated secondary Ab for 2 h, and again washed 3 times for 10 min. Chemiluminescent signal was detected using the ECL detection kit (ThermoFisher Scientific, 32,106) and a MicroChemi 4.2 device (DNR Bio Imaging System). For quantification, Gel-analyzer Software 2010a was used.

### Immunocytochemistry

Immunostainings were performed as previously described [13]. Cells were fixed with 4% paraformaldehyde (containing 10% sucrose) for 7 minutes and incubated for two hours using a blocking solution (PBS + 10% Goat Serum + 0.2% Triton X‐100). The same solution was used for the incubation with primary antibodies for 24 h at 4 °C. After incubation with primary antibodies, three washes with PBS were performed before incubating the cells with secondary antibodies (diluted 1:1,000 in PBS) for two hours at room temperature. Afterward, cells were washed three times again and mounted with ProLong^TM^ Gold Antifade Mountant with DAPI (Thermo Fisher Scientific, P36935) or with ibidi Mounting Medium (Ibidi, 50001).

### Microscopy and image analysis

Fluorescence microscopy was performed with a Thunder imaging system (Leica) equipped with a DFC9000 sCMOS camera, an HC PL Fluotar 20× air (N.A. 0.4) objective, and using the LasX software (Leica).

Confocal microscopy was performed by using a laser-scanning microscope (Leica DMi8) equipped with an ACS APO 63X oil DIC immersion objective. Images were acquired using the LasX software (Leica), with a resolution of 1024 × 1024 pixels and a number of Z-stacks (step size of 0.3 μm).

Images were analyzed by using the ImageJ 2.14.0 software. To analyze the intensity levels of nuclear phospho-c-Jun^Ser63^ in immunostaining, the Z-stack was collapsed with the maximum intensity projection tool of ImageJ. A region of interest (ROI) was drawn using the MAP2 and DAPI channels as a reference to the nucleus and the mean intensity of phospho-c-Jun^Ser63^ signal was measured.

To analyze the intensity of P62/SQSTM1, the Z-stack was collapsed, a region of interest (ROI) was drawn around the cell soma using the MAP2 channel as a reference and the mean intensity was measured.

Aggresomes were detected using the PROTEOSTAT*®* Aggresome detection kit (Enzo, ENZ-51035-0025). To analyze the somatic aggresome area, the Z-stacks were also collapsed, and a ROI was drawn around the cell soma using the MAP2 channel as a reference. Afterwards, a threshold was set for the PROTEOSTAT*®*/aggresome, and the area above the threshold was measured.

To analyze the size of MN somata, a ROI was drawn around the soma using the MAP2 channel as a reference. The area of the ROI was then converted into µm^2^. Primary dendrites were counted manually in the MAP2 channel.

To analyze the intensity of HOMER and Bassoon, the Z-stacks were also collapsed and a ROI was drawn around the primary dendrites, approximately 20 µm far from the soma, using the polygon selection of ImageJ. The MAP2 channel was used as a reference.

To identify synaptic contacts, colocalization between HOMER (postsynaptic marker) and Bassoon (presynaptic marker) was performed using Imaris 9.7.0 (Bitplane). At first, the MAP2 channel was used to draw a surface of reference with the Surface tool. Afterward, the puncta for HOMER and Bassoon were detected semi-automatically in the respective channel (with the Spots tool). Between HOMER and Bassoon, a minimum distance of 0.8 μm between the center of the respective spots and a maximum distance of 1 μm from the dendrite was accepted as an interaction, which could be interpreted as a synapse.

The same computational parameters and post-acquisition adjustments were used to analyze images from the same differentiation and to image display within the figures.

### Antibody list

The primary antibodies used in experiments with hiPSC-derived MNs were: anti-MAP2 (Encor, CPCA-MAP2; diluted 1:1000), Proteostat® aggresome detection kit (Enzo, ENZ-51035-0025; diluted 1:5000), anti-phospho-c-Jun (Ser63) (Cell Signaling, 91,952; diluted 1:1000), anti-phospho SQSTM1/p62 (Abcam, 56416; diluted 1:1000), anti-Homer1 (Synaptic Systems, 160 003; diluted 1:500), anti‐Bassoon (Enzo, ADI‐VAM‐PS003‐D; diluted 1:500), anti-Neurofilamet heavy (Abcam, 8135, diluted 1:10,000) and anti-β-Actin (Sigma-Aldrich, A5316, diluted 1:25,000).

For immunostainings, the following secondary antibodies from Thermo Fisher Scientific were used at 1:1000 dilution: anti-Chicken Alexa Fluor® 488 (A-11039), goat anti-Rabbit Alexa Fluor® 568 (A-11036), goat anti-Guinea Pig Alexa Fluor® 568 (A-11075), goat anti-Mouse Alexa Fluor® 647 (A-21235).

For Western blot experiments, the secondary HRP-conjugated anti-Mouse (1:3000 dilution) and anti-Rabbit (1:1000 dilution) antibodies from DAKO were used.

### qRT-PCR

Total RNA from hiPSC-derived MN was extracted using the RNeasy Mini kit (Qiagen, 74104) following the instructions from Qiagen. First-strand synthesis and quantitative real-time-PCR amplification were performed in a one-step using the QuantiFast™ SYBR Green RT-PCR kit (Qiagen, 208054) in a total volume of 20 µl. The primers used for qRT-PCR were purchased (Qiagen QuantiTect Primer Assays, Qiagen-validated primers without sequence information). The following settings were used: 10 min at 55 °C and 5 min at 95 °C, followed by 40 cycles of PCR for 5 s at 95 °C for denaturation and 10 s at 60 °C for annealing and elongation (one-step). The SYBR Green I reporter dye signal was measured against the internal passive reference dye (ROX) to normalize non-PCR-related fluctuations. The Rotor-Gene Q software (version 2.0.2) was used to calculate the cycle threshold values. *GAPDH* expression levels were used to normalize resulting data.

### Animals

High-copy B6SJL-Tg (SOD1*G93A)1Gur/J mice were obtained from Jackson Laboratory and bred with B6/SJL WT females (also from Jackson); the colony was maintained as previously reported [19] and locally bred under standard housing conditions. Transgenic and WT C57BL/6JRj mice were group-housed according to their genotype, with ad libitum access to water and food and a day-night cycle of 12 h. Only male mice were used in the current study. Animal experiments were performed at Ulm University in compliance with institutional guidelines (Tierforschungszentrum, Ulm) for organ removal (license no. o.217-9) when mice were 35 days old. At this time point, transgenic mice do not show clear signs of degeneration [20, 21], allowing the analysis of the complete pool of spinal MNs.

### Laser microdissection of FF MNs

Mice were anesthetized with 1 mg/kg body weight ketamine chlorhydrate and 0.5 mg/kg xylazine. Once deep anesthesia was confirmed by the absence of a toe-pinch response, the chest cavity was carefully exposed and a precise incision was made in the right atrium using sharp forceps. Subsequently, the left ventricle was gently infused with ice-cold phosphate-buffered saline (PBS) at a controlled flow rate of 5-7 ml/min over a period of 2 min using a peristaltic pump. Finally, spinal cord samples were quickly dissected, embedded in OCT (TissueTek), and stored at −80 °C.

After sterilizing all the necessary equipment under UV light, 12 μm-thick cryosections were cut at −20 °C and mounted on RNase-free Polyethylene naphtalate (PEN) membrane slides. Sections were fixed in 70% ethanol diluted in DEPC-H_2_O at −20 °C and stained with 1% cresyl violet in 50% ethanol/DEPC-H_2_O for 1 min each. Then, slides were incubated for 1 min in 70% and 100% ethanol at +4 °C. Then, 30 ventrolateral motoneurons per experimental group were microdissected and captured using the Laser Microdissection System (Palm MicroBeam, Zeiss) on a 500 µL clear adhesive cap (Carl Zeiss).

Cells were lysed by adding 21 µl 1× SuperScript III first-strand RT buffer (Invitrogen) containing 1% NP40 at 42 °C for 20 min. Reverse transcription was carried out using the SuperScript III First-Strand Synthesis System for RT-PCR kit (Invitrogen). Briefly, 50 ng/µL random hexamers and 10 mM dNTP mix were added and incubated at 65 °C for 5 min. Then, the cDNA synthesis mix (10× RT buffer, 25 mM MgCl_2_, 0.1 M DTT, RNaseOUT 40 U/µl, SuperScriptIII RT 200 U/µl) was added and incubated for 10 min at 25 °C followed by 50 minutes at 50 °C. The reaction was terminated at 85 °C for 5 min. To remove excess RNA, RNase H was mixed to the solution and incubated for 20 min at 37 °C.

### RNA fluorescent in situ hybridization (FISH)

To investigate the expression of *SYT13* in the murine spinal cord, we used tissue samples from 3-month-old WT mice. Afterward RNAscope in situ hybridization was performed using the RNAscope^®^ Multiplex Fluorescent Reagent Kit v2 (ACD‐BIO, 323100).

Briefly, slides were incubated in H_2_O_2_ for 10 min at RT. Afterward, they were washed with distilled water 5 times. The Target Retrieval was brought to boiling (at 100 °C) using a Thermoblock. The slices were now placed in a container at 100 °C and incubated for 15 min with the boiling Target Retrival. Now the slides were transferred to 100% Ethanol for 3 min. Afterwards, they were left to dry at RT (approx. 5–10 min). To create a hydrophobic barrier a ImmEdge® Hydrophobic Barrier PAP Pen (Vector Laboratories, H-4000) was used, and left to dry overnight or RT. The dried slides were loaded into the ACD EZ Batch Slide holder, and 5 drops of Protease III were added to each section and incubated in a HybEZ^TM^ II Oven at 40 °C for 30 min. At this point, cells were hybridized with pre‐warmed RNAscope™ Probe- Mm-Syt13 (ACD‐BIO, 582091) in the HybEZ^TM^ II Oven at 40 °C for 4,5 h. After hybridization, slides were incubated with the amplification buffers AMP1 for 30 min at 40 °C, AMP2 for 30 min at 40 °C, and AMP3 for 15 min at 40 °C.

Between each incubation step, the slides were washed twice for 5 min at room temperature with the 1× Wash Buffer provided with the kit. Afterwards, the slides were treated with HRP-C1 for 15 min at 40 °C and washed again 3 times for 5 min at room temperature with the 1× Wash Buffer. Now a treatment with Opal 570 reagent (SKU FP1488001KT diluted 1:2000 in TSA buffer) for 30 min at 40 °C was performed. Samples were then washed three times with Wash Buffer, incubated with HRP‐Blocker reagent for 15 min at 40 °C, washed again twice at room temperature with the × Wash Buffer for 5 min, and processed for immunostaining as described above.

Images were acquired by confocal microscopy and were quantified as described above.

For these experiments, MNs were identified by using the neuronal markers NeuN (Synaptic Systems 173 004) and CHAT (Abcam rb181023). The Z-stacks were collapsed, and a region of interest (ROI) was drawn around the cell soma. To quantify the *SYT13* RNA foci, the FindFoci plugin of ImageJ was used. The diameter of the MN was determined by using the measurement tool of ImageJ.

### Data analysis

Differential expression analysis of the RNAseq data was performed using the DESeq2R package (1.20.0). The resulting *P*-values were adjusted using the Benjamini and Hochberg’s approach for controlling the false discovery rate. Genes with an adjusted *P*-value < 0.05 found by DESeq2 were assigned as differentially expressed. For Gene Set Enrichment Analysis (GSEA), genes were ranked according to the degree of differential expression in the different samples, and then the predefined Gene Set were tested to see if they were enriched at the top or bottom of the list.

The prediction of the transcription factors controlling the expression of the genes commonly altered in the SYT13^+/−^ transcriptome and ALS spinal cord samples was performed with the TTRUST (version2) database [22].

The correlation of SYT13^+/−^ and gene expression signatures was performed with the SigCom LINCS tool developed by the Ma´ayan Laboratory [23] by searching for SYT13 (Fig. 4G, H) and uploading the up- and down-regulated transcripts in SYT13^+/−^ vs SYT13^+/+^ cultures (Fig. 4I).

The PCA based on the expression of bona fide TP53 targets was based on genes identified using “The TP53 Database” (https://tp53.isb-cgc.org) and identified in the RNAseq dataset generated in this manuscript.

To compare two independent groups (genotypes) in western blot, immunocytochemistry, and qPCR, an unpaired t-test with Welch correction in the case of normally distributed data and a nonparametric Mann–Whitney test for non-normal distribution was used (GraphPad Prism, Version 10.1.1).

### Generalized linear modeling and machine learning analysis of answer ALS data set

RNA-seq data used in the preparation of this article were obtained from the ANSWER ALS Data Portal (AALS-01184) [24]. For up-to-date information on the study, visit https://dataportal.answerals.org. We included expression data from 122 female ALS patients, 50 female controls, 218 male ALS patients, and 51 male controls, totaling 441 samples. The dependent class variables of ALS were set to 0, and controls were set to 1. A generalized linear model was created using glm (Class ~ SYT13 + Sex + SYT13:Sex, data = df1, family = binomial), which determined a significant interaction between SYT13 and Sex. Several machine learning models were tested using the automated STREAMLINE package [25] on the same cohort of female and male ALS and control patient data. All SYT gene homologues as well as sex were input features. The sex class variables of female were set to 0, and male were set to 1. Briefly, the default settings for STREAMLINE were run: Phase 1: Exploratory Analysis and Phase 2: Data Preprocessing to count instances, determine missing data, cleaning, feature engineering, and partitioning of training and test data sets; Phase 3: Feature Importance (FI) Evaluation using MultiSURF to determine feature interaction, Phase 4: Feature Selection based on FI, Phase 5: Modeling with all available algorithms, Phase 6: Statistics Summary and Figure Generation to evaluate average cross-validation and statistical comparisons of each algorithm performance.

### Next generation sequencing

The used samples were analyzed using Next generation sequencing (Illumina: TruRisk™ Panel). The sequencing was performed using NextSeq High Output Kit v2.5 (300Cycles). The data were analyzed using the Software Varvis version 1.25.0 (Limbus Medical Technologies GmbH, Rostock) and filtered for the gene *TP53* (NM_000546.6). The sequence data were evaluated in comparison to the respective reference sequence (hg19, NCBI). Identified variants were compared against different databases and filtered based on allele frequency (MAF < 1%).

### Multiplex ligation-dependent probe amplification (MLPA)

To assess possible deletions or duplications within the gene *TP53*, the used samples were analyzed using the MPLPA-Kit P056-D1 from MRC-Holland according to the manufacturing protocol. The result was compared to three different control samples. The data were analyzed using the Software Sequence Pilot from JSI medical systems

## Results

### SYT13 reduction is associated with ALS in male subjects

**Fig. 1:**
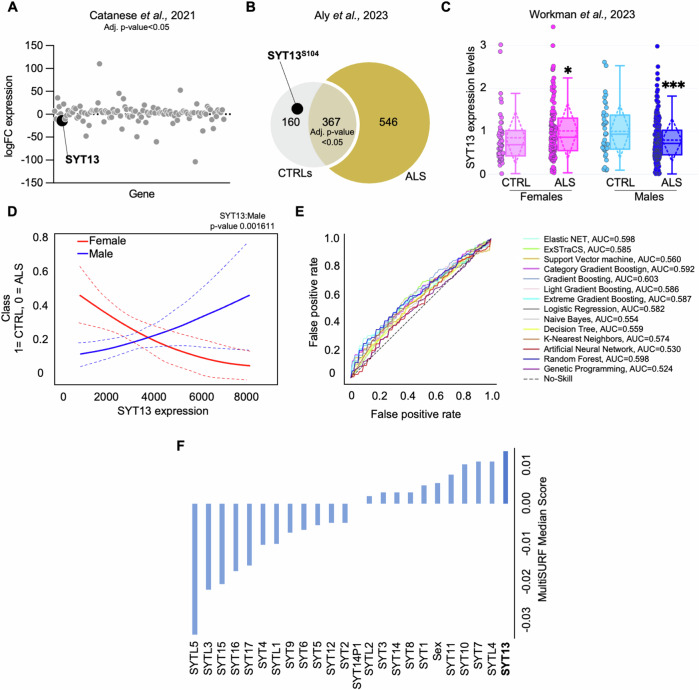
Reduced levels of *SYT13* correlate with disease outcome in male patients. **A** *SYT13* expression levels are significantly reduced in the transcriptome of C9orf72-mutant MNs. *N* = 2 control and ALS lines (*n* = 6 replicates for each genotype). **B** Phospho-SYT13 is only detected in the proteome of control MNs, and not in ALS. *N* = 3 CTRLs and *N* = 8 ALS patients. **C** *SYT13* is differentially expressed in females and male subjects: female ALS patients have higher levels of the synaptotagmin than sex-matched controls, whose *SYT13* expression is lower than their male counterparts. In contrast, MNs derived from male patients show lower abundance of *SYT13* transcript than healthy individuals. *N* = 101 CTRLs (50 females and 51 males) and 340 = ALS (122 females and 218 males). Box and whiskers represent Mean ± SD. **D** Linear regression model highlighting a significant correlation between the *SYT13* levels and disease outcome. **E** Sex and expression levels of synaptotagmins do not yield significant prediction models using different machine learning pipelines. **F** MultiSURF algorithm highlights that SYT13 has the most relative feature importance associated with ALS.

In the attempt to identify cellular mechanisms determining MN vulnerability, we considered omic datasets previously published by our group and found a converging downregulation of SYT13 in ALS-related human MNs at both RNA and protein levels. Specifically, the expression levels of *SYT13* mRNA were significantly lower in MNs carrying the C9orf72 pathogenic GGGGCC hexanucleotide repeat expansion (HRE) than in cultures obtained from healthy and isogenic controls (Fig. 1A) [18]. In line with this evidence, the phosphorylated form of SYT13 (serine 104) was detected only in the MN samples obtained from healthy controls and not from heterogeneous ALS patients with *C9orf72, FUS, TBK1* and *TARDBP* mutations (Fig. 1B) [13]. Given the limited number of hiPSC lines used in our in-house datasets, we aimed at confirming the reduced expression of *SYT13* in a broader cohort of ALS patient-derived cultures. To this end, we analyzed the transcriptome data available through the AnswerALS consortium, which includes more than 400 lines [26]. Since previous analysis performed with this dataset highlighted sex as a major contributor to gene expression variability in ALS, we looked at the levels of *SYT13* in females and males separately. Interestingly, we noted that hiPSC-derived neurons obtained from male healthy controls had significantly higher levels of *SYT13* transcript than females. Moreover, female patients showed higher *SYT13* mRNA than their sex-matched controls, while the expression of this specific synaptotagmin in male patients was significantly lower than in healthy individuals (Fig. 1C). Accordingly, we could demonstrate the presence of a significant interaction between the expression of SYT13 and sex (males) affecting ALS outcome by using a general linear model (GLM) (Fig. 1D), while the same correlation had an opposite trend in females but did not reach the threshold for statistical significance.

We then investigated whether the effect driven by SYT13 was specific for this member of the synaptotagmin family and tested several machine learning pipelines using the expression pattern of all SYT homologs along with sex as variables. This approach did not yield any good predictive model for disease outcome based on the levels of the different synaptotagmins (Fig. 1E). However, analysis of relative feature importance using the multiSURF algorithm [27] highlighted *SYT13* as the gene with the highest degree of interaction with other features in predicting regardless of ALS phenotype, implying a strong expression relationship of this family of genes with *SYT13* (Fig. 1F).

### Vulnerable fast fatigable motor neurons express low levels of Syt13

Since the physiological expression of *SYT13* seems to determine vulnerability in different MN subpopulations [15], we performed fluorescent in situ hybridization to assess its levels in the spinal cord of WT mice. Spinal MNs were identified as NeuN-CHAT double-positive cells within the ventral horn and, by analyzing the number of *Syt13* RNA foci, we noticed a significant, inverse correlation between the levels of *Syt13* and the size of the MNs considered. Since the fast-fatigable (FF) represent the most vulnerable population of spinal motor neurons in ALS and are characterized by a larger size than slow-firing (S) ones [28, 29], we set a size threshold to distinguish between these two subpopulations and found that MNs with somata larger than 30 µm had significantly less RNA foci than smaller ones (Supplementary Fig. 1A). This suggested that, within the heterogeneous population of motor neurons, the FF ones are characterized by the lowest levels of *Syt13*. In fact, a recent single-cell transcriptome analysis of the murine spinal cord highlighted lower levels of *Syt13* in FF (expressing *Kcnq5*) than in other MNs [30]. To evaluate the meaning of these results in the context of ALS, we performed laser-capture microdissection to isolate FF MNs from the spinal cord of p35 SOD1^G93A^ mice and WT littermates [31]. This early time point allowed us to comprehensively analyze the spinal MN population under disease conditions since, at this age, SOD1 mice still do not show signs of muscle denervation and MN loss [10]. The expression levels of *Syt13* were then assessed by qPCR and, in support of its putative role in determining neuronal vulnerability, we found that the FF MNs isolated from early-stage mutant mice displayed a strong, close to significance (*p*-value = 0.0676) down-regulation of this transcript than WT animals (Supplementary Fig. 1B).

### SYT13^+/−^ motor neurons are characterized by a time-dependent appearance of neurodegenerative phenotypes

**Fig. 2:**
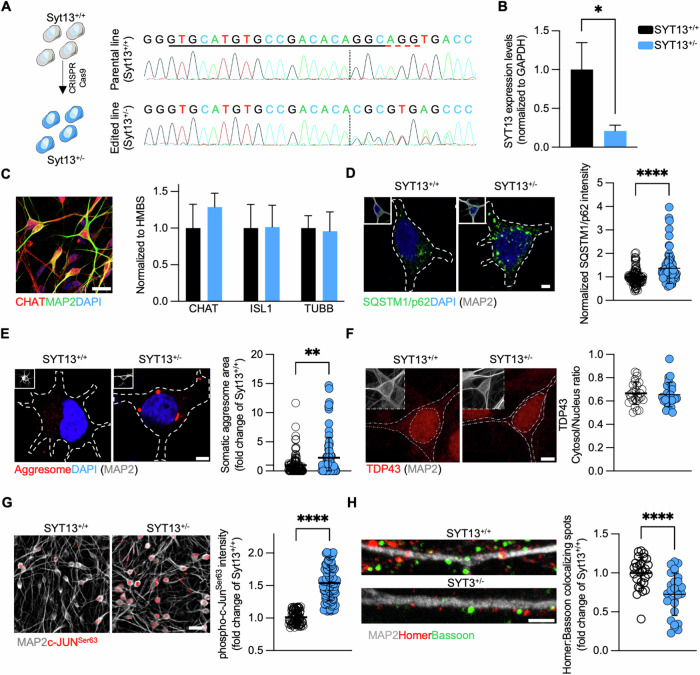
Heterozygous loss of *SYT13* triggers typical ALS phenotypes in 5-weeks old human MNs. **A** Schematic representation and sequencing of the CRISPR-Cas9 strategy to generate the SYT13^+/−^ hiPSC line. **B** Rt-qPCR of the *SYT13* levels in SYT13^+/−^ and SYT13^+/+^ MNs. *N* = 6. *p < 0.05. **C** Representative image of DIV21 hiPSC-derived MNs positively stained against CHAT and MAP2. The expression levels of typical neuronal markers are not altered SYT13^+/−^ MNs at this stage of culture. Scale bar 20 μm. **D** DIV35 SYT13^+/−^ MNs have higher levels of SQSTM1/p62 than their isogenic controls. Scale bar 5 μm. *N* = 75 MNs from 3 independent differentiations. ****p < 0.0001. **E** Accumulation of aberrant cytosolic aggresomes is also detected in DIV35 SYT13^+/−^ MNs. Scale bar 5 μm. *N* = 75 MNs from 3 independent differentiations. **p < 0.01. **F** SYT13^+/−^ MNs do not show signs of TDP43 pathology. Scale bar 5 μm. Scale bar 5 μm. *N* = 55 MNs from 3 independent differentiations. Data are represented as the mean ± SD. **G** The phosphorylation levels of the stress marker Jun are significantly higher in SYT13^+/−^ MNs than SYT13^+/+^ ones. Scale bar 20 μm. *N* = 90 MNs from 3 independent differentiations. ****p < 0.0001. **H** Excitatory synapses are reduced in SYT13-deficient neurons. Scale bar 3 μm. *N* = 30 MNs from 3 independent differentiations. ****p < 0.0001. Data are represented as the mean ± SD.

In line with the neuroprotective role of SYT13 overexpression in models of MN diseases, our data suggested a crucial function of this poorly investigated protein in determining sufferance in the most vulnerable subpopulation of alpha-motor neurons. Thus, we set out to reveal the specific cell-autonomous mechanisms linked to SYT13 deficiency. We used CRISPR-Cas9 technology to heterozygously knockout *SYT13* (SYT^+/−^) in the hiPSC line GM23338, which was generated by reprogramming of fibroblasts from a caucasian 55 years-old male. DNA sequencing of edited and parental hiPSCs identified the successful deletion of two guanines in the *SYT13* coding region of the engineered line (Fig. 2A). We then efficiently differentiated hiPSCs from both genotypes into spinal motor neurons (Supplementary Fig. 2A) and, even though SYT13^+/−^ cells had a significantly lower expression of *SYT13* than wild-type ones (Fig. 2B), we did not detect any significant alteration in the expression of typical MN markers between cultures of both genotypes at *day* in vitro (DIV) 21 (Fig. 2C). According with the maintained differentiation capacity, 3-weeks-old SYT13^+/−^ cultures did not display any significant sign of cellular sufferance: the levels of the autophagy receptor SQSTM1/p62 (Supplementary Fig. 2B), which forms toxic aggregates in ALS MNs [18], as well as the stress marker c-Jun (Supplementary Fig. 2C), whose increased activation is a shared phenotype across the ALS spectrum [13], were indeed comparable to those of SYT13^+/+^ neurons. Interestingly, we could detect an aging-dependent appearance of ALS-related phenotypes as MNs were cultured longer: 5-weeks-old SYT13^+/−^ cultures showed an aberrant accumulation of SQSTM1/p62 aggregates (Fig. 2D) and of cytotoxic, perinuclear aggresomes (Fig. 2E), without displaying any sign of pathological cytoplasmic accumulation of TDP43 (Fig. 2F). According to the increased stress linked to the accumulation of toxic protein aggregates, aged SYT13^+/−^ MNs showed a significantly increased phosphorylation of c-Jun (Fig. 2G) and a detrimental loss of synaptic contacts (Fig. 2H). All in all, this data demonstrated a progressive appearance of degenerative phenotypes in human motor neurons with reduced levels of SYT13.

### RNA sequencing of SYT13^+/−^ human motor neurons highlights an ALS-like transcriptional landscape

**Fig. 3:**
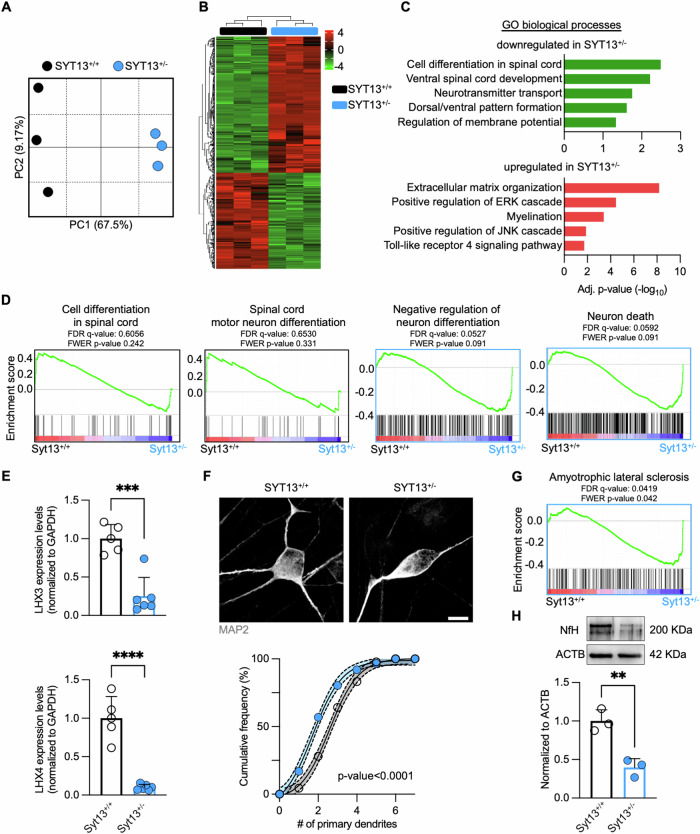
The SYT13^+/−^ transcriptome is characterized by impaired MN development and increased stress response. **A** PCA displaying the transcriptional separation between SYT13^+/−^ and SYT13^+/+^ MNs. **B** Heatmap showing the differentially expressed genes in SYT13^+/−^ cultures. **C** Representative GO biological processes terms obtained by enrichment analysis based on the SYT13^+/−^ transcriptome. **D** GSEA plots showing processes most related to SYT13^+/+^ or SYT13^+/−^ neurons. **E** Confirmation of *LHX3* and *LHX4* expression in SYT13^+/−^ MNs with rt-qPCR. *N* = 5 differentiations. **F** SYT13^+/−^ MNs have fewer primary dendrites than isogenic controls. Scale bar 10 μm. *N* = 3 differentiations. **G** GSEA plot showing significant correlation between the SYT13^+/−^ transcriptome and ALS ontology. **H** Western blot analysis showing reduced levels of NfH in SYT13^+/−^ cultures. *N* = 3 differentiations. *p < 0.05. Data are represented as the mean ± SD.

We then performed RNA sequencing (RNAseq) to comprehensively analyze the alterations linked to *SYT13* deficiency in human MNs. Principal component analysis (PCA) highlighted a net separation along the PC1 between the transcriptomes of SYT13^+/+^ and SYT13^+/−^ cells (Fig. 3A), which indicated the heterozygous deletion of *SYT13* as the major contributor to the transcriptional variance observed. By looking at the differentially expressed genes (DEGs) between the two genotypes, we identified 678 down- and 477 upregulated genes in SYT13^+/−^ cultures (Fig. 3B; Supplementary Table 1A). Downregulated transcripts highlighted significant alterations in spinal cord development, patterning, and neurotransmitter transport, while the upregulated genes enriched in terms linked to extracellular matrix and myelination, as well as ERK, JNK, and toll-like receptor pathways (Fig. 3C; Supplementary Table 1B). Additionally, gene set enrichment analysis (GSEA) supported the impairment in processes of spinal cord differentiation, as the transcriptome of SYT13^+/−^ MNs was significantly associated with negative neuronal differentiation and cellular death (Fig. 3D). Moreover, we confirmed by qPCR the downregulation of the *LHX3* and *LHX4* genes (Fig. 3E) in SYT13^+/−^ neurons, which also displayed a significantly lower number of primary dendrites in comparison to their SYT13^+/+^ counterpart (Fig. 3F). Thus, this transcriptional data highlighted that *SYT13* deficiency deeply impairs the correct development of spinal MNs, which end up in showing signs of sufferance reminiscent of those observed in ALS patients. Accordingly, the transcriptome of SYT13^+/−^ cultures significantly correlated with the Amyotrophic Lateral Sclerosis annotation (Fig. 3G) and was characterized by significantly lower levels of the biomarker NfH than SYT13^+/+^ ones (Fig. 3H).

### The SYT13^+/−^ transcriptome converges toward ALS and pro-apoptotic signatures

**Fig. 4:**
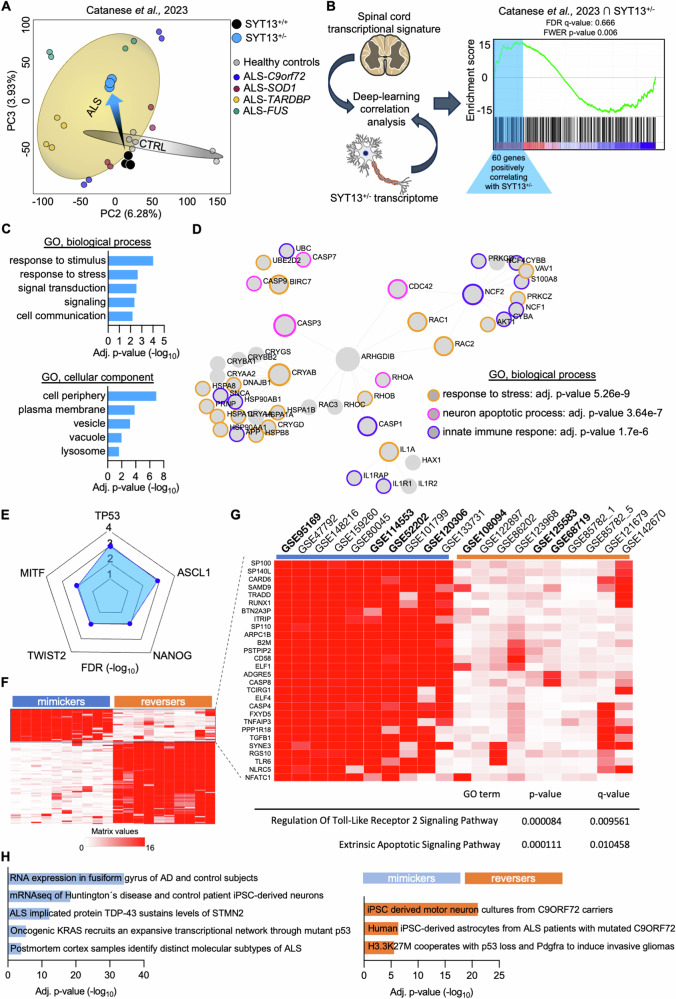
The SYT13^+/−^ transcriptional fingerprint correlates with ALS signatures and highlights TP53 activation. **A** PCA plot integrating the SYT13^+/−^ transcriptome with an in-house RNAseq [32] performed with ALS mutants and healthy MNs. Reduced levels of *SYT13* are sufficient to shift the transcriptional program of human MNs within the ALS centroid. **B** Machine learning-based integrative analysis of the SYT13^+/−^ and ALS spinal cord transcriptome identifies 60 genes positively correlating with both signatures. **C** Enrichment analysis based on the 60 genes highlighted by the integrative transcriptome analysis. **D** Gene network and enrichment based on the transcripts commonly shared by the SYT13^+/−^ and ALS transcriptomes highlights stress, inflammation, and pro-apoptotic terms. **E** TP53 is the transcription factor mostly associated with the gene cluster shared by the SYT13^+/−^ and ALS transcriptional signatures. **F** Heatmap displaying the top 10 mimicker and reverser signatures identified with the SigCom LINCS algorithm and associated with SYT13. **G** The genes of the top mimicker transcriptomes are linked to toll-like receptor signaling and apoptosis, linking SYT13 and ALS signatures. **H** Transcriptomes that significantly correlate with the SYT13^+/−^ signature are associated with ALS, neurodegeneration, and TP53.

**Fig. 5:**
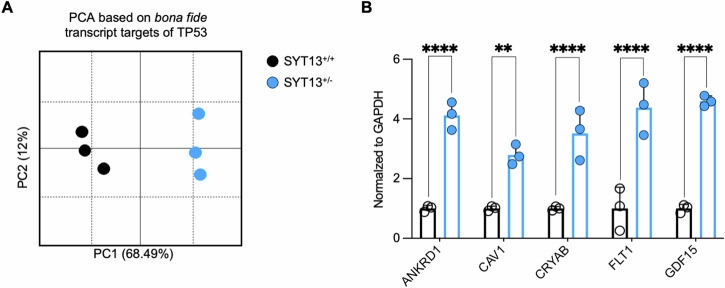
The transcriptional program linked to TP53 separates SYT13^+/−^ MNs from their isogenic control. **A** PCA plot generated using the expression of 242 TP53 target genes and displaying a clear separation between SYT13^+/−^ and SYT13^+/−^ transcriptomes along PC1. **B** Single-tube qPCRs confirming the higher expression of five TP53 targets in SYT13^+/−^ MNs than in isogenic controls. *N* = 3. **p < 0.01, ****p < 0.0001. Data are represented as the mean ± SD.

Since the RNAseq analysis highlighted a striking resemblance of ALS features when the levels of *SYT13* are reduced in MNs, we confronted the SYT13^+/−^ transcriptome with the one of mutant MNs obtained from patients with mutations in *C9orf72, FUS, TARDBP*, and *SOD1* genes [32]. After removal of PC1 from both datasets to eliminate batch effect, we could still observe a separation between control cultures and the heterogenous pool of ALS mutants. While the SYT13^+/+^ samples clustered, as expected, close to the other controls, we noticed that the heterozygous deletion of *SYT13* was sufficient to shift the samples at the center of the ALS centroid (Fig. 4A), indicating a strong similarity between the disease transcriptional program and *SYT13* deficiency. To identify these transcriptional similarities in the context of disease progression, we then used a machine learning approach to integrate the SYT13^+/−^ transcriptome with the signature obtained from *post-mortem* samples of ALS patients [32] and identified 60 genes (Supplementary Table 1C) significantly correlating with both datasets (Fig. 4B). The transcripts identified with this approach were mainly related to stress response pathways as well as vesicular/lysosomal structures (Fig. 4C), and network analysis highlighted some important molecules involved in neuronal cell death (such as members of the caspase family) and of the immune response (like interleukins and heat-shock proteins) as major determinants of the overlap between SYT13^+/−^ and ALS signatures (Fig. 4D). Interestingly, the expression of these genes was predicted to be mostly controlled by the transcription factor TP53 (Fig. 4E), whose activation contributes to the neurodegenerative processes associated with ALS [33, 34]. Since we did not identify any sequence abnormality within the *TP53* gene in the CRISPR-edited lines by using Next generation sequencing and multiplex ligation-dependent probe amplification, these findings suggested a pathological convergence toward the activation of this specific transcription factor between ALS and reduced levels of SYT13. Accordingly, we found that the expression of *SYT13* was significantly altered in transcriptional signatures (Fig. 4F; Supplementary Table 1D) linked to TP53 activation and ALS, and the top up-regulated genes linked to mimicker signatures of SYT13 were involved in toll-like receptor signaling and pro-apoptotic pathway (Fig. 4G). Furthermore, the link between altered levels of *SYT13*, TP53 activation, and neuronal sufferance was strengthened by the significant correlation between the SYT13^+/−^ transcriptome and RNAseq signatures of neurodegeneration and TP53 activity (Fig. 4H; Supplementary Table 1E). To further strengthen these findings, we looked at high-confidence targets of TP53 within our SYT13^+/−^ RNAseq. With this approach, we found 242 genes in our dataset (Supplementary Table 1F) whose expression was sufficient to effectively separate in PCA the SYT13^+/−^ from the SYT13^+/+^ transcriptome (Fig. 5A), confirming the relation between SYT13 deficiency and the TP53-related transcriptional program. These data were further strengthened by single-tube qPCR experiments performed with independent samples, which highlighted significantly higher levels of the *ANKRD1, CAV1, CRYAB, FLT1*, and *GDF15* transcripts in SYT13^+/−^ cultures than in isogenic ones.

In conclusion, reduced expression of *SYT13* is sufficient to trigger alterations in the transcriptional program linked to TP53, which contributes to cellular sufferance and vulnerability of human motor neurons.

## Discussion

Even though the major pathologies that detrimentally affect the central nervous system (CNS) are continuously and deeply investigated, the exact mechanisms behind the preferential vulnerability of specific neuronal populations and/or brain areas in different disorders have not been clarified yet. Humans can in fact suffer from a large and heterogeneous number of neurological conditions, which differ from each other in terms of onset, symptoms, lethality and, at the cellular level, pathobiochemistry. This applies also to fatal neurodegenerative diseases, which are mostly characterized by the accumulation of toxic protein species and progressive loss of neurons [35]. Here the underlying heterogeneity is not only represented by the specific neuronal populations affected, but also by the nature of protein aggregates characterizing the different forms of neurodegeneration. In the case of ALS, the specific cellular vulnerability within the neurons of the motor system is also extremely puzzling. While both upper and lower MNs being affected in this disease, the corticofugal theory of pathology progression suggests reduced vulnerability within the motor cortex, where pathology begins but lesser signs of degeneration are observed, than in the spinal cord [36, 37]. In addition, the different subtypes of spinal motor neurons appear to be differentially affected during the disease stages. Evidence obtained mostly from animal models suggests that the FF MNs are the first to degenerate already at the pre-symptomatic phase, while S ones display higher resistance and reduced signs of degeneration [38]. Since these MN subpopulations display distinct firing properties, it has been suggested that differences in electrophysiological parameters and excitability might determine differential vulnerability [10, 39, 40]. Still, neuronal activity is a complex and finely-tuned process that is controlled through different cellular mechanisms, including dynamic changes of gene expression and synaptic inputs [41]. For these reasons, finding an exhaustive explanation to the molecular determinants of MN vulnerability has been extremely challenging until now. A common pathological feature displayed by vulnerable MNs in ALS and SMA is represented by pre-synaptic alterations, where especially the molecular machinery of vesicle release, which includes SNAREs and synaptotagmins, appears to be mostly altered [13, 42, 43]. Interestingly, these synaptic aberrations do not appear to simply be the consequence of neurons dying within a network, since reduced levels of synaptic transcripts can be detected already before neuronal loss [32]. This indicates that the neuronal transcriptome might contain crucial information to at least partially reveal the principles of intrinsic vulnerability. The physiological expression of selected gene pools might indeed determine the basis of susceptibility in neurons which, in association with specific pathogenic mutations, eventually degenerate. Interestingly, this seems to apply also when looking at the transcriptome patterns observed in male and female ALS patients, whose motor neurons (as well as brain and spinal cord samples) are characterized by distinct signatures [26]. In this context, the sexually dimorphic baseline expression of *SYT13* in healthy female and male MNs as well antithetical dysregulation in ALS female and male motor neurons suggest a role for SYT13 in influencing an increased disease risk in males. Given that males predominate among ALS patients under age 65 [44] and transcriptomic analysis of hiPSC-derived MNs revealed much stronger ALS signatures in males [26], this suggests that iPSC models of MNs can reflect the earliest molecular events leading up to ALS pathobiology. In fact, the male-enriched transcripts observed in ALS mainly cluster in pro-inflammatory and apoptotic pathways [26]. The increased expression of SYT13 in healthy male MNs compared to healthy female MNs reflects intrinsic differences between sexes, where male MNs require higher SYT13 activity. Interestingly, male ALS conditions downregulate SYT13, and this putatively renders them more vulnerable to downstream degeneration. In contrast, female ALS conditions are able to upregulate SYT13 as a protective, compensatory response to an as-of-yet undefined upstream promoter of ALS pathogenesis. Future investigations into SYT13 specifically in female ALS conditions will be needed to test this hypothesis. Even though reducing the higher incidence of ALS and, at least in some cohorts [45], SMA in men to the reduced expression of a single gene surely represents a simplistic view of the disease complexity, it is still reasonable to consider that the sex-dependent transcriptional differences might play a crucial role in defining cellular susceptibility to disease. But what is the role of *SYT13* in the context of cellular stress and death? Despite belonging to a protein family mainly involved in vesicle release, the lack of a calcium-binding domain in the structure of this synaptotagmin suggests a different function than the one of most of the other family members [46]. In fact, while SYT13 has been linked to vesicle trafficking and protein internalization in pancreatic cells [16], direct evidence for its involvement in the typical release of neurotransmitters [47] has not been provided yet. In contrast, SYT13 has been associated with several tumors where it promotes malignant cellular proliferation by controlling the Akt pathway [48]. In this context, repression of *SYT13* inhibits tumor growth and induces apoptosis in colorectal tumor cells [49]. This provides evidence in support of an additional and still uncharacterized function of this protein in controlling cellular proliferation and differentiation, likely also in the case of post-mitotic cells as neurons. Our data are indeed in agreement with this theory, as the SYT13^+/−^ transcriptome was characterized by a significant down-regulation of genes involved in the patterning and differentiation of the spinal cord such as the LHXs [50]. This suggests that the reduced *SYT13* levels might impact the physiological differentiation of MNs first (as supported by their reduced dendritic complexity), leading to subsequent neuronal sufferance mediated by the activation of TP53. Notably, this transcription factor is strongly linked to both SMA and ALS [33, 34, 51], which are also characterized by altered neurodevelopmental features [52, 53]. On the basis of these considerations and of the striking transcriptional phenotype displayed by SYT13^+/−^ MNs, it is somehow surprising that this gene has not been causally linked to major motor neuron diseases. A possible explanation is that loss of function (LoF) variants in SYT13 have a Loss-of-Function observed/expected upper bound fraction (LOEUF) score of 0.54 (range 0.38–0.78), suggesting low tolerance. Accordingly, homozygous *Syt13* KO mice are not viable [16]. Nevertheless, the Project MinE Data Browser lists one ALS patient with a LoF variant in this gene, which anyway occurred together with a typical GGGGCC repeat expansion in the *C9orf72* gene, thus wiping away the possibility of pathogenicity for the *SYT13* mutation. Interestingly, this patient was previously mentioned in a case report for some atypical disease features such as a bulbar onset at 42 years of age and co-occurrence of multiple sclerosis symptoms [54]. This might suggest that *SYT13* variants, when tolerated, might act as disease modifiers rather than being pathogenic per se. In this direction, more attention should be dedicated to the frequency of rare genetic variants with unknown clinical significance, as they might reveal important aspects of the pathology that have been overseen until now. In the specific case of this work, our data revealed important and novel aspects of motor neuron vulnerability associated with SYT13 and strengthened the idea that interventions aiming at increasing its expression in these specific cells might represent a valid therapeutic approach for motor neuron diseases such as SMA and ALS.

### Supplementary information

Supplementary figures

Supplementary table

Uncropped western blot related to Figure 3H

## Data Availability

The RNAseq data are available at Gene Expression Omnibus (GEO) under the accession number GSE261848.
