# Supplementary material for: Heterozygous knockout of Synaptotagmin13 phenocopies ALS features and TP53 activation in human motor neurons
Source: Cell Death Dis. 2024 Aug 3;15(8):560. doi: 10.1038/s41419-024-06957-3 (PMC11297993; doi:10.1038/s41419-024-06957-3)
Supplement: Supplementary file 1 — Supplementary figures [file 41419_2024_6957_MOESM1_ESM.pdf]

Heterozygous knockout of Synaptotagmin13 phenocopies ALS features and TP53 activation in human motor neurons

Johannes Lehmann[1,*], Amr Aly[1,*], Christina Steffke[1,2], Luca Fabbio[1], Valentin Mayer[1], Natalie Dikwella[2], Kareen Halablab[2], Francesco Roselli[2,3], Simone Seiffert[4], Tobias M Boeckers[1,3], David Brenner[2,3], Edor Kabashi[5], Medhanie Mulaw[6,#], Ritchie Ho[,7,8,9,10,#], Alberto Catanese[1,3,5,#]

[1] Institute of Anatomy and Cell Biology, Ulm University School of Medicine, 89081 Ulm, Germany

[2] Department of Neurology, Ulm University School of Medicine, 89081 Ulm, Germany

[3] German Center for Neurodegenerative Diseases (DZNE), Ulm Site, Germany

[4] Institute of Human Genetics, Ulm University and Ulm University Medical Center, 89081, Ulm, Germany.

[5] Institut Imagine, University Paris Descartes, Necker-Enfants Malades Hospital, 75015 Paris, France.

[6] Unit for Single-Cell Genomics, Medical Faculty, Ulm University, 89081 Ulm, Germany

[7] Center for Neural Science and Medicine, Cedars-Sinai Medical Center, Los Angeles, CA 90048, USA

[8] Board of Governors Regenerative Medicine Institute, Cedars-Sinai Medical Center, Los Angeles, CA 90048, USA

[9] Department of Biomedical Sciences, Cedars-Sinai Medical Center, Los Angeles, CA 90048, USA

[10] Department of Neurology, Cedars-Sinai Medical Center, Los Angeles, CA 90048, USA

* equal contribution

[#] Co-senior authors

Correspondence: alberto.catanese@uni-ulm.de

This file contains 2 Supplementary Figures

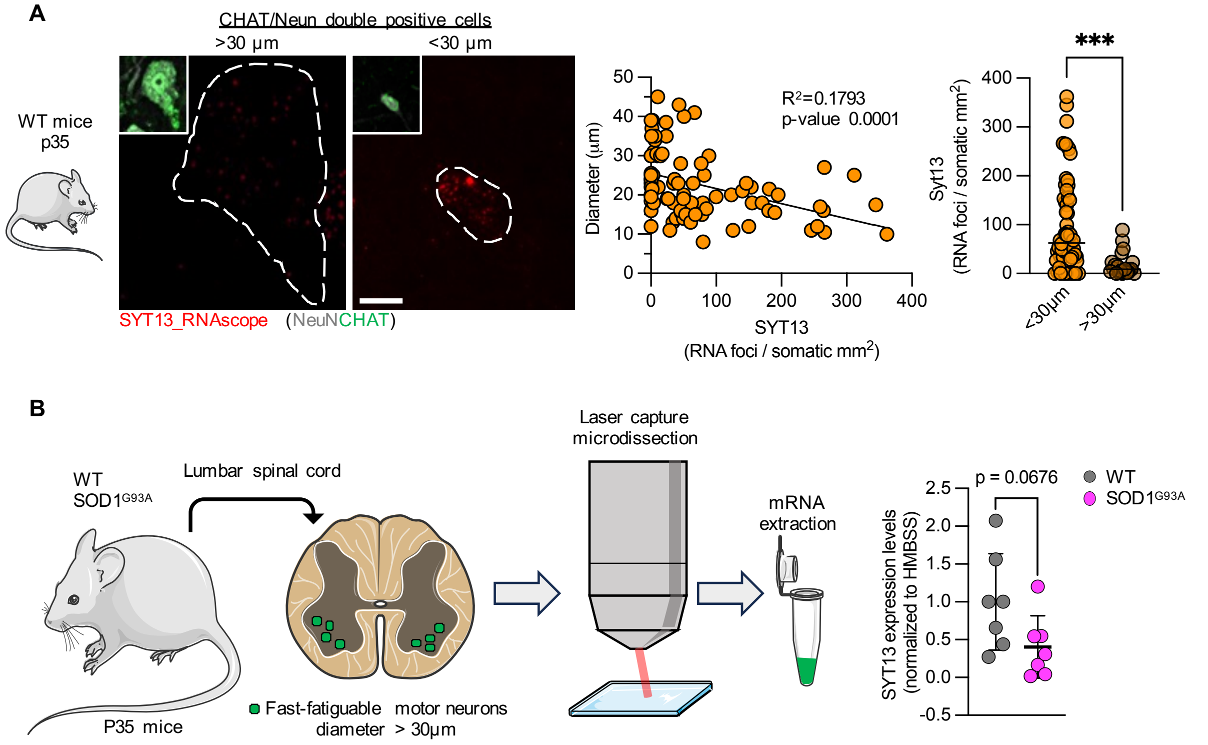

Supplementary Figure 1

(A) Representative confocal images of large (diameter > 30µm) and small (diameter < 30µm) motor neurons in the ventral horn of lumbal spinal cord sections of p35 WT mice. The larger (presumably FF) MNs have reduced *SYT13* foci than smaller ones. Scale bar 10 µm. N = 87 MNs from 2 mice. * $p < 0.001$.

(B) Schematic representation of the workflow to isolate mRNA from FF MNs of WT and $SOD1^{G93A}$ mice through laser microdissection. N = 7. p = 0.0676.

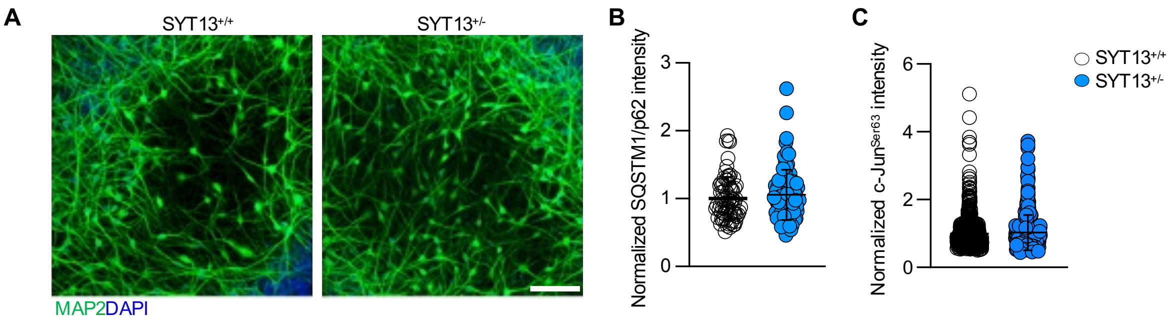

Supplementary Figure 2

(A) Representative confocal images of SYT13$^{+/+}$ and SYT13$^{+/-}$ cultures positively stained against MAP2 at DIV21. Scale bar 50 µm.

(B) 3-weeks old SYT13$^{+/-}$ MNs do not show accumulation of SQSTM1/p62.

(C) The levels of phospho-cJun are comparable in DIV21 SYT13$^{+/+}$ and SYT13$^{+/-}$ MNs.
