# Supplementary figures and images for: Heterozygous knockout of Synaptotagmin13 phenocopies ALS features and TP53 activation in human motor neurons
Source: Cell Death Dis. 2024 Aug 3;15(8):560. doi: 10.1038/s41419-024-06957-3 (PMC11297993; doi:10.1038/s41419-024-06957-3)

kDa

ACTIN BETA

250

130

100

70

55

35

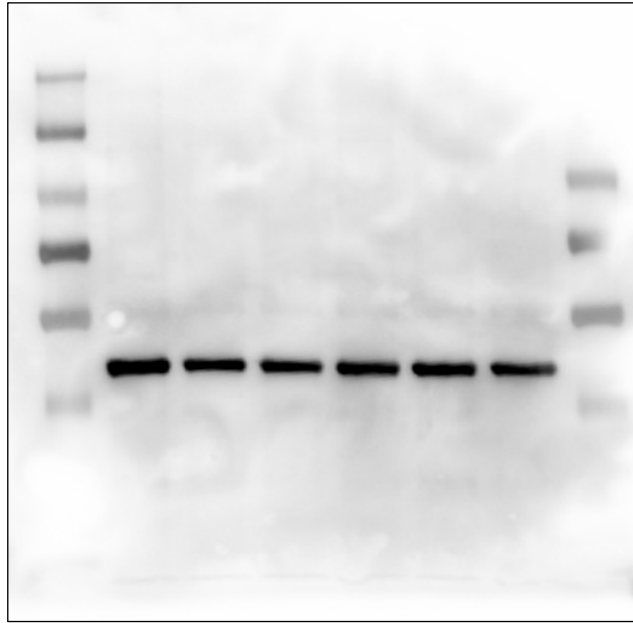

NEUROFILAMENT HEAVY CHAIN

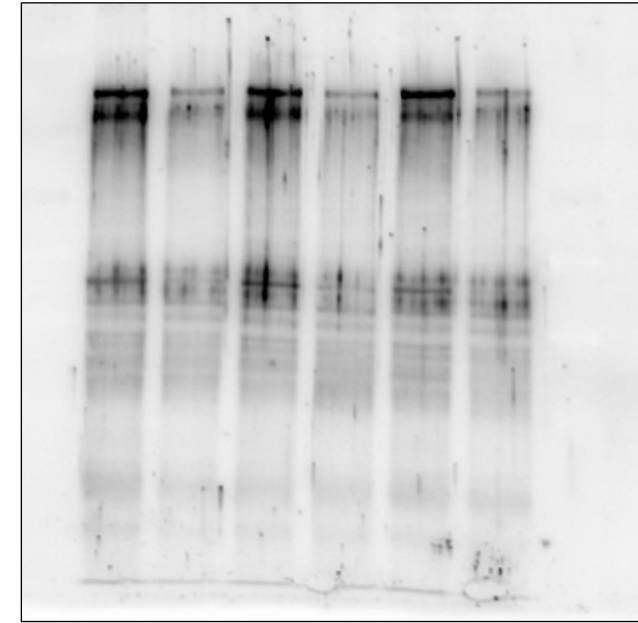

Supplement: Supplementary file 3 — Uncropped western blot related to Figure 3H [file 41419_2024_6957_MOESM3_ESM.pdf]
